# Peritoneal Tuberculosis Mimicking Pseudomyxoma Peritonei: A Diagnostic Challenge

**DOI:** 10.7759/cureus.97283

**Published:** 2025-11-19

**Authors:** Siddhi Chawla, Mohith Belagihalli Venkatesh, Chhagan L Birda, Divya Aggarwal, Mahaveer Singh Rodha

**Affiliations:** 1 Trauma and Emergency, Radiodiagnosis, All India Institute of Medical Sciences, Jodhpur, IND; 2 Diagnostic and Interventional Radiology, All India Institute of Medical Sciences, Jodhpur, IND; 3 Gastroenterology, All India Institute of Medical Sciences, Jodhpur, IND; 4 Pathology, All India Institute of Medical Sciences, Jodhpur, IND; 5 Trauma and Emergency, Surgery, All India Institute of Medical Sciences, Jodhpur, IND

**Keywords:** cbnaat, computed tomography, malignancy mimic, peritoneal tuberculosis, pseudomyxoma peritonei

## Abstract

Peritoneal tuberculosis (TB) is a rare extrapulmonary manifestation that can closely mimic peritoneal carcinomatosis or pseudomyxoma peritonei (PMP) due to overlapping imaging features such as omental caking, ascites, and adnexal masses. We present a case of a young woman in her early 20s with peritoneal TB with scalloped liver margins and peritoneal thickening with bulky ovaries, initially suspected as PMP. Imaging and laboratory findings led to suspicion of malignancy, but cartridge-based nucleic acid amplification test (CBNAAT) analysis confirmed TB. This case underscores the importance of recognizing imaging clues that might help to distinguish TB from peritoneal neoplasms.

## Introduction

Tuberculosis (TB) remains a major global health concern, with extrapulmonary TB (EPTB) comprising 10-42% of reported TB cases globally, especially among immunocompetent individuals in endemic regions such as India, which accounts for approximately 26% of the global TB burden [1]. Peritoneal tuberculosis is a rare manifestation of EPTB, accounting for 1-2% of all TB cases and 4-10% of abdominal TB cases, and poses a considerable diagnostic challenge due to its nonspecific clinical and radiological features [2-4].

Peritoneal tuberculosis results from infection by *Mycobacterium tuberculosis*, most commonly secondary to reactivation of latent peritoneal foci or spread from primary sites such as the lungs, gastrointestinal tract, or genitourinary system. The bacilli may reach the peritoneum via hematogenous dissemination, direct extension from infected organs or lymph nodes, or rupture of a tuberculous focus in the bowel or fallopian tube. The ensuing granulomatous inflammation leads to exudative ascites, peritoneal thickening, and nodularity, explaining the diverse imaging patterns observed [2-4].

Patients often present with vague symptoms, such as abdominal distension, pain, fever, anorexia, or ascites, which can closely resemble presentations of advanced intra-abdominal malignancies like pseudomyxoma peritonei (PMP) or peritoneal carcinomatosis [3-5]. Radiologically, PMP -- usually caused by mucinous tumors of the appendix or ovary -- is characterized by gelatinous ascites, scalloping of visceral surfaces, and peritoneal implants. These features may be misinterpreted in cases of tuberculous peritonitis, especially in young women [6]. Additionally, elevated cancer antigen 125 (CA-125), often used as a biomarker for ovarian cancer, can also rise in TB due to mesothelial irritation, further complicating the clinical picture [5].

Early and accurate differentiation between these conditions is critical to avoid unnecessary surgical interventions. Imaging modalities such as contrast-enhanced computed tomography (CECT) and magnetic resonance imaging (MRI) play a key role, but their specificity is limited. Integration of radiologic findings with endoscopic ultrasound (EUS), microbiological assays, histopathology, and ascitic fluid analysis remains essential in reaching the correct diagnosis [4].

This case highlights the diagnostic dilemma of peritoneal TB mimicking PMP in a young immunocompetent woman and underscores the importance of multimodal evaluation essential to prevent misdiagnosis and unnecessary surgical interventions.

## Case presentation

A previously healthy young woman in her early 20s presented with a two-month history of progressive abdominal distension, intermittent lower abdominal pain, anorexia, and low-grade fever. She denied weight loss, gastrointestinal or urinary complaints, menstrual irregularities, or previous pelvic surgery. There was no history of TB contact, immunosuppression, or chronic illness.

**Figure 1:**
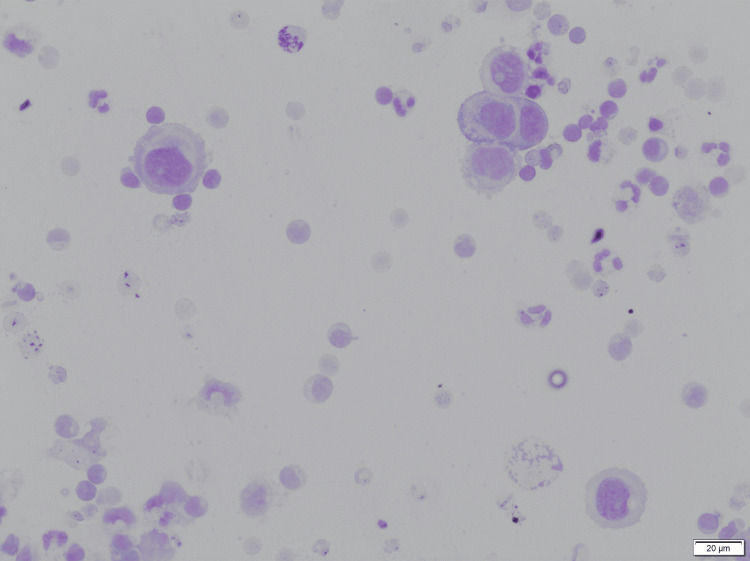
Cytological analysis of ascitic fluid shows reactive mesothelial cells with background lymphomononuclear cells (Giemsa stain, 200x).

On examination, she was afebrile and hemodynamically stable. Abdominal examination revealed distension with a positive fluid thrill, diffuse tenderness, and abdominal pain with exaggeration of tenderness on palpation associated with bloating, suggesting peritoneal irritation, but there were no palpable masses or peripheral lymphadenopathy. Initial laboratory investigations (Table 1) showed normocytic anemia (hemoglobin 9.0 g/dL), elevated aspartate aminotransferase (50.6 U/L), hypoalbuminemia (3.27 g/dL), and hyperglobulinemia (4.89 g/dL). Ascitic fluid analysis revealed exudative ascites with high protein content (6.64 g/dL), low glucose (64 mg/dL), and albumin of 2.87 g/dL. Tumor markers, including alpha-fetoprotein (AFP), carcinoembryonic antigen (CEA), and CA 19-9, were within normal limits; however, CA-125 was modestly elevated at 142 U/mL. The cartridge-based nucleic acid amplification test (CBNAAT) for *Mycobacterium tuberculosis* from ascitic fluid was negative. Cytological analysis revealed reactive mesothelial cells without malignant features (Figure 1).

**Table 1:** Summary of the key laboratory and ascitic fluid findings, highlighting exudative ascites with mild transaminase elevation, hypoalbuminemia, and elevated CA-125. CBNAAT: cartridge-based nucleic acid amplification test; CA-125: cancer antigen 125; CA 19-9: cancer antigen 19-9.

Parameter	Observed value	Reference range
Hemoglobin	9.0 g/dL	12–16 g/dL (female)
Aspartate aminotransferase (AST)	50.6 U/L	10–40 U/L
Albumin	3.27 g/dL	3.5–5.0 g/dL
Globulin	4.89 g/dL	2.0–3.5 g/dL
Ascitic fluid protein	6.64 g/dL	<2.5 g/dL (transudate); >2.5 g/dL (exudate)
Ascitic fluid glucose	64 mg/dL	70–100 mg/dL
Ascitic fluid albumin	2.87 g/dL	-
Alpha-fetoprotein (AFP)	Within normal limits	<10 ng/mL
Carcinoembryonic antigen (CEA)	Within normal limits	<5 ng/mL
CA 19-9	Within normal limits	<37 U/mL
CA-125	142 U/mL	<35 U/mL
CBNAAT for Mycobacterium tuberculosis	Negative	Negative

**Figure 2:**
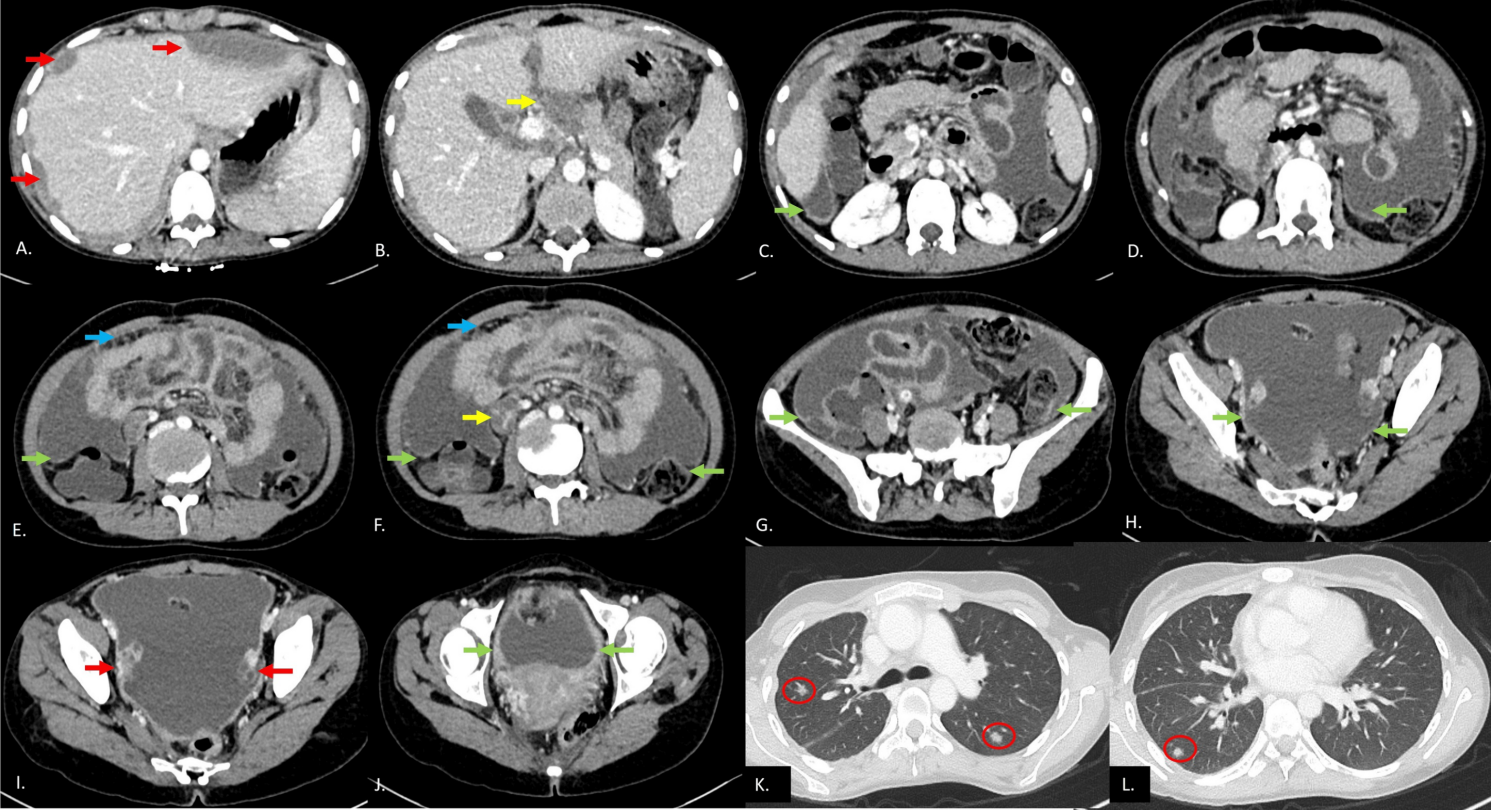
Sequential axial CECT abdomen images shows (A) scalloping of liver margins due to subcapsular collections in liver (red arrows), (B) heterogeneously enhancing hypodense lesion in region of porta infiltrating the body of pancreas (yellow arrow), (C)-(J) smooth peritoneal thickening (green arrows in (C)-(J)) with mild omental thickening (blue arrow in (E)) and necrotic mesenteric nodes (yellow arrow in (F)) and bilateral bulky ovaries (right > left) (red arrows in (I)). (K) and (L) Axial lung window sections show focal nodular consolidations in bilateral lung (red circles). CECT: contrast-enhanced computed tomography.

**Figure 3:**
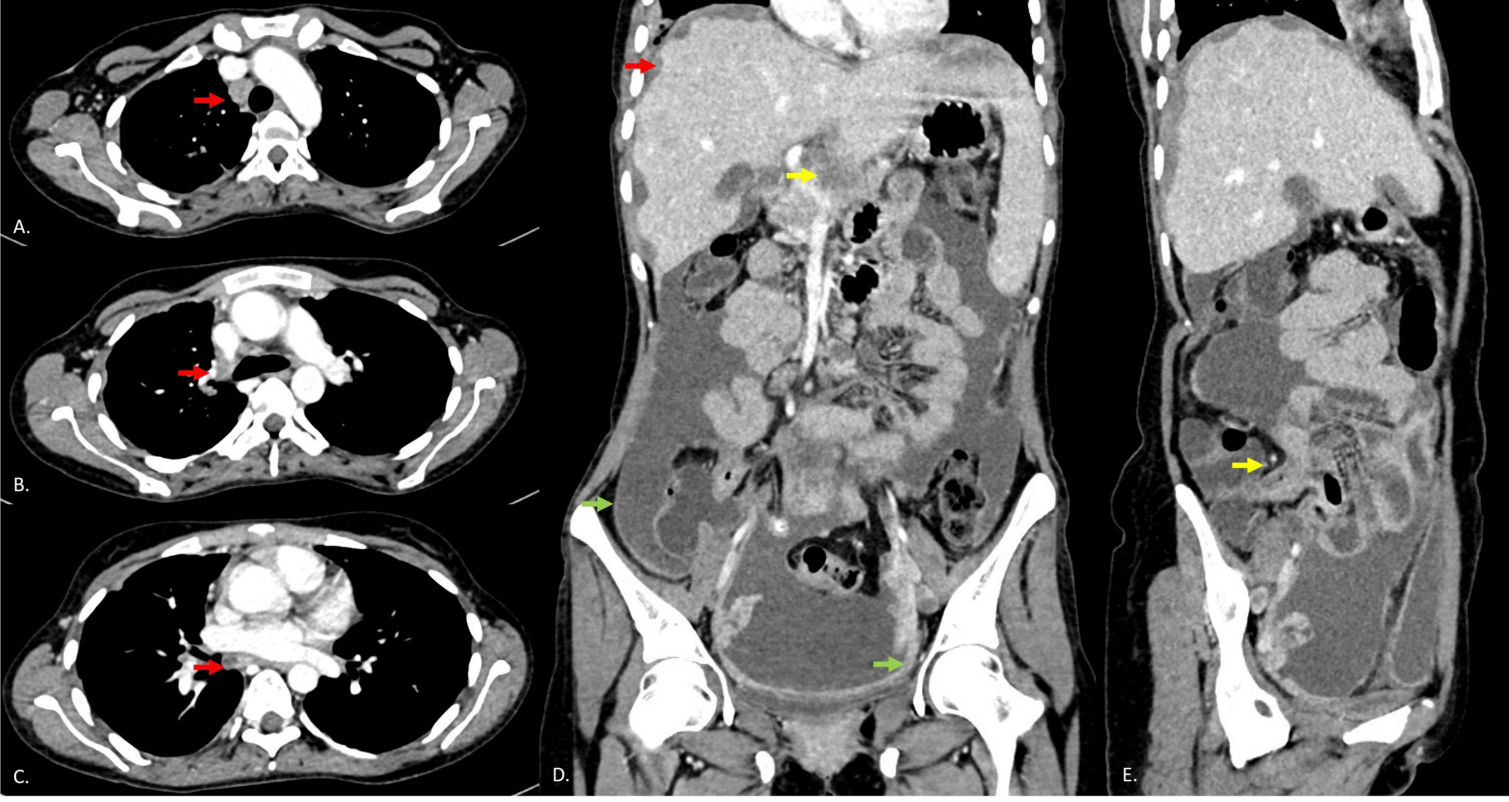
Sequential axial CECT thorax images (A)-(C) show multiple enlarged heterogeneous mediastinal lymph nodes (red arrows). (D) Additional coronal images of abdomen show scalloped appearance of liver margin (red arrow), periportal hypodense lesion infiltrating pancreas (yellow arrow) with smooth peritoneal thickening (green arrow). (E) Oblique sagittal images show thickened ileocecal junction (yellow arrow). CECT: contrast-enhanced computed tomography.

Abdominal ultrasonography showed mild splenomegaly, moderate ascites with internal septations and echogenic debris, and bilaterally bulky ovaries without defined masses. Contrast-enhanced computed tomography (CECT) of the abdomen (Figures 2, 3) demonstrated moderate ascites, smooth peritoneal and omental thickening, and scalloping of hepatic margins due to hypodense lesions. Multiple enlarged conglomerated nodes were seen in periportal, peripancreatic, and mesentery locations. A large hypodense heterogeneously enhancing lesion, likely nodal conglomerate, appeared to involve the pancreatic body. There was diffuse mural thickening with stratification of the terminal ileum, and bilateral ovaries were bulky without an obvious mass lesion within them. CECT thorax (Figures 2, 3) further revealed patchy consolidation in bilateral lung fields with nodular pleural thickening, minimal right pleural effusion, and mediastinal lymphadenopathy.

These findings raised a strong suspicion for disseminated tuberculosis, with differentials including pseudomyxoma peritonei (PMP)/peritoneal carcinomatosis (PC) due to primary ovarian/pancreatic lesions, mucinous neoplasm of the pancreas.

**Figure 4:**
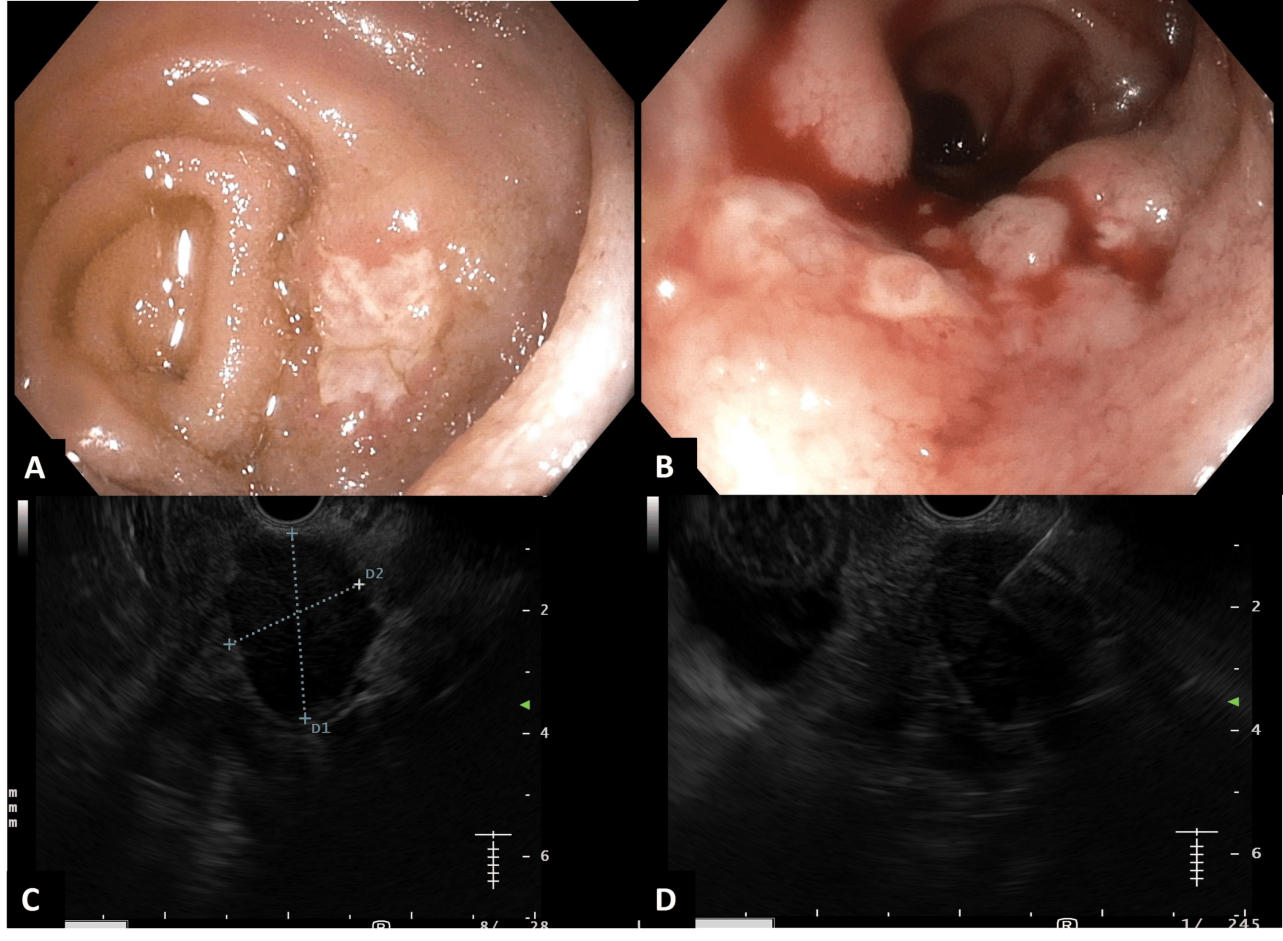
Colonoscopy image shows (A) superficial, round ulcers in the terminal ileum. (B) Nodular mucosal changes are also seen in the terminal ileum. (C) Endoscopic ultrasound (EUS) reveals a large, hypoechoic, oval lymph node in the peripancreatic region. (D) Fine-needle biopsy is obtained under EUS guidance.

**Figure 5:**
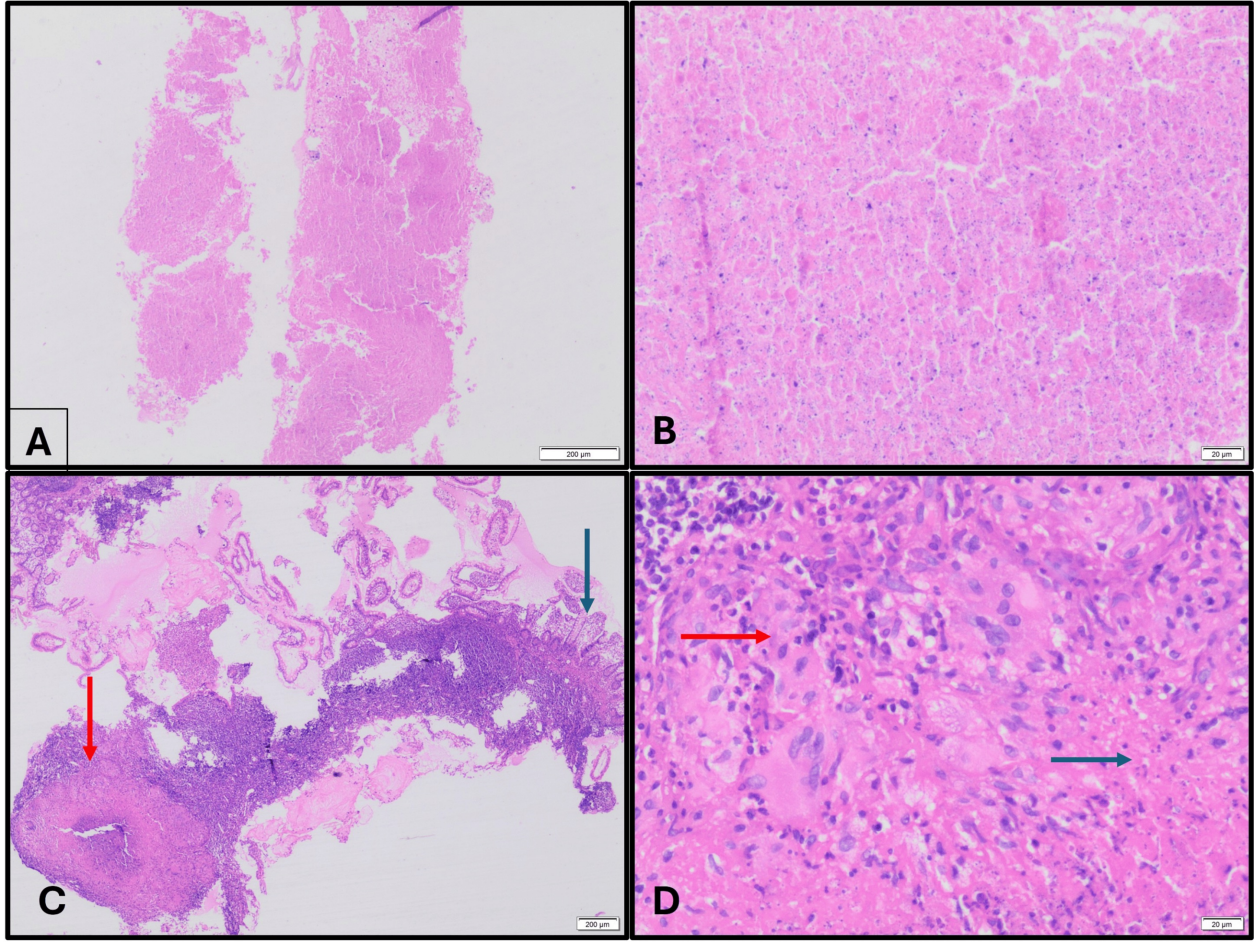
EUS-guided fine-needle biopsy from periportal lymph node shows (A) low-power histological image of a periportal lymph node shows necrotic areas (hematoxylin and eosin (H&E), 20×). (B) High-power view confirms extensive necrosis (H&E, 200×). (C) Ileal biopsy reveals preserved villous architecture (blue arrow) with deeper zones showing necrotizing granulomatous inflammation (red arrow) (H&E, 20×). (D) Higher magnification shows necrosis (blue arrow) and epithelioid granulomas (red arrow) (H&E, 400×). EUS: endoscopic ultrasound.

To confirm the diagnosis, endoscopic ultrasound-guided fine-needle biopsy (EUS-FNB) of the periportal lymph nodes was performed, revealing necrotizing granulomatous inflammation. Colonoscopy showed multiple circumferential ulcers and nodular mucosa in the terminal ileum. Biopsies from these lesions also demonstrated necrotizing granulomatous inflammation, although Ziehl-Neelsen staining for acid-fast bacilli was negative. Meanwhile, ascitic fluid adenosine deaminase (ADA) was also elevated (41 U/L), and repeat CBNAAT from the tissue sample was positive for *Mycobacterium tuberculosis* (Figures 4, 5).

The constellation of imaging findings, elevated ascitic fluid ADA, histopathological evidence of granulomatous inflammation, CBNAAT positivity of tissue biopsy, normal tumor markers with mildly raised CA-125 levels, and exclusion of primary malignancy on imaging supported a diagnosis of disseminated tuberculosis involving the peritoneum, periportal lymph nodes, terminal ileum, and pancreas. The patient was started on standard four-drug anti-tubercular therapy (isoniazid, rifampicin, pyrazinamide, and ethambutol) for two months, followed by isoniazid and rifampicin for an additional four months. She showed clinical improvement during hospitalization and was discharged to follow-up in the outpatient department.

## Discussion

Peritoneal tuberculosis remains a major diagnostic challenge due to its ability to clinically and radiologically mimic intra-abdominal malignancies such as peritoneal carcinomatosis (PC) or pseudomyxoma peritonei (PMP). This is particularly relevant in TB-endemic countries where diagnostic delays may lead to inappropriate interventions, including unnecessary surgeries and chemotherapy. The diagnostic overlap is even more complex in young women, where elevated CA-125 levels and adnexal lesions can misleadingly suggest ovarian carcinoma or PMP.

Peritoneal TB presents in three classic forms: wet (ascitic), dry (fibrous adhesions), and fibrotic-fixed (mass-like lesions with matted bowel loops). The wet form is most common and can closely resemble PMP due to ascites, peritoneal thickening, and omental involvement. Imaging findings often overlap, and a careful evaluation of ancillary features is critical for accurate diagnosis. In peritoneal TB, ascites is usually straw-colored and proteinaceous and may demonstrate septations on ultrasound or CT. CECT often reveals smooth peritoneal thickening, central clumping of bowel loops, and diffuse omental thickening. In contrast, PMP is characterized by dense mucinous ascites causing scalloping of visceral surfaces, particularly the liver and spleen, along with nodular peritoneal deposits and possible calcifications [1,4,7].

In our patient, subcapsular hepatic fluid collections mimicked the scalloped liver margins seen in PMP. However, the absence of calcifications and the presence of smooth rather than nodular omental thickening favored TB. Furthermore, prominent necrotic periportal lymphadenopathy, which is typical of TB than malignancy, guided us toward targeted EUS-guided biopsy, which revealed granulomatous inflammation.

The presence of adnexal lesions can further complicate the differential diagnosis. Tubercular salpingitis typically presents as bilateral hydrosalpinges or pyosalpinges, while PMP-related adnexal masses are large, complex, solid-cystic lesions with differential attenuation due to differences in protein content within the locules of mucin-containing ovarian lesions. Other malignancies of mucinous origin associated with PMP can be primary pancreatic or appendiceal. Our patient demonstrated bilateral bulky ovaries (measuring 4.2 x 3.5 cm on the right side and 4.5 x 3.6 cm on the left side with normal ovarian architecture and enhancing surface deposits) with no discrete primary ovarian mass, a finding more in line with tubercular etiology [2,3].

Serum CA-125 levels are frequently elevated in both tuberculosis and malignancy. However, in tuberculosis, this elevation reflects mesothelial inflammation rather than neoplastic transformation. Simsek et al. demonstrated that CA-125 levels in tuberculous peritonitis may overlap with those in malignancy, with a mean value of 316.6 IU/mL in their study. Although the levels are generally lower in tuberculosis and should not independently trigger surgical exploration, especially in TB-endemic areas [5], Bae et al. proposed a threshold of approximately 1000 U/mL (specificity ~92%) to help differentiate ovarian malignancy from tuberculous peritonitis; however, significant overlap limits its diagnostic reliability [6]. Our patient had mildly elevated CA-125 levels but no other serological or imaging evidence of malignancy, supporting a more conservative diagnostic approach.

Ascitic fluid analysis remains a cornerstone in differentiating peritoneal TB from PMP. In TB, ascites is exudative, lymphocyte-predominant, and low serum ascites albumin gradient (SAAG). However, early cases of TB or concurrent infection may present with neutrophil predominance, leading to diagnostic confusion [7,8]. Adenosine deaminase (ADA) levels are often elevated in TB ascites and, while not specific, they support the diagnosis in conjunction with other findings. CBNAAT testing demonstrates high specificity (~98%) but limited sensitivity in ascitic fluid, particularly in paucibacillary disease. In our case, the peritoneal fluid tested negative for CBNAAT; however, a repeat test performed on the tissue sample was positive, highlighting the importance of appropriate sampling in suspected cases of tuberculosis. A positive CBNAAT result is confirmatory for TB [9-11].

MRI offers additional diagnostic utility in pelvic tuberculosis, demonstrating heterogeneous, septated ascites and peritoneal thickening, unlike the homogeneous mucinous signal of pseudomyxoma peritonei on T2-weighted imaging. Diffusion-weighted imaging aids further distinction, showing restricted diffusion in inflammatory collections. Wu et al. and Naeem et al. emphasized MRI's role in characterizing peritoneal and genitourinary TB, with reported diagnostic accuracy of approximately 85-90%, though interpretation requires experience and context [4,8].

The gold standard for diagnosis remains histopathological confirmation via peritoneal biopsy. However, in selected cases with supportive clinical, radiological, and microbiological evidence, as in our patient, unnecessary laparoscopy may be avoided. EUS-guided sampling of necrotic nodes and colonoscopic biopsy from the ileocecal junction revealed granulomatous inflammation consistent with TB, obviating the need for surgical intervention or empirical chemotherapy. 

Lastly, follow-up with imaging and CA-125 levels also provides valuable differentiation between peritoneal tuberculosis and peritoneal carcinomatosis. In tuberculosis, successful anti-tubercular therapy leads to a marked reduction in ascites, resolution of septations, and regression of peritoneal thickening or nodularity within weeks to months with a fall in CA-125 levels. In contrast, peritoneal carcinomatosis typically shows persistent or progressive peritoneal deposits, increasing ascitic volume and CA-125 levels, or development of new implants despite therapy. Thus, interval improvement on follow-up MRI or CT strongly favors an infectious over a malignant etiology [12].

Thus, early diagnosis and initiation of anti-tubercular therapy are vital to prevent complications and unnecessary treatments. Our case highlights the importance of a multidisciplinary, context-aware diagnostic approach, integrating imaging, laboratory data, and minimally invasive sampling to accurately distinguish TB from its malignant mimic, pseudomyxoma peritonei [1,4,7,10]. Table 2 summarizes the imaging and diagnostic features differentiating peritoneal TB and pseudomyxoma peritonei. 

**Table 2:** Imaging and diagnostic features differentiating peritoneal TB and pseudomyxoma peritonei. CBNAAT: cartridge-based nucleic acid amplification test; AFB: acid-fast bacilli; TB: tuberculosis.

Feature	Peritoneal tuberculosis	Pseudomyxoma peritonei
Ascitic fluid	Straw-colored, septated [3,4]	Mucinous, dense; scalloping of visceral surfaces [3]
Omental appearance	Diffuse thickening [4,8]	Nodular mucin deposits; calcified masses [3]
Peritoneal thickening	Smooth, uniform [4,9]	Irregular, nodular implants [3]
Adnexal mass	Bilateral hydrosalpinx [2,7,8]	Unilateral/bilateral mucinous ovarian tumor; appendiceal or pancreatic cystic lesion [3]
Lymphadenopathy	Common; often necrotic nodes [4,7,8]	Rare [3]
CA-125	Mild to moderately elevated [5,7]	Often markedly elevated [3,6]
CT findings	Omental caking, septated ascites [4,8,9]	Scalloped organs, mucin lakes [3]
MRI findings	Hydrosalpinx; peripheral rim enhancement [8]	High T2 signal mucin; solid-cystic masses [3]
Confirmatory test	CBNAAT, AFB culture [1,10,11]	Histopathology; mucin cytology [3]

## Conclusions

Peritoneal tuberculosis remains a great mimicker, often closely resembling pseudomyxoma peritonei and other peritoneal malignancies, particularly in young women presenting with ascites, adnexal masses, and elevated CA-125 levels. High clinical suspicion, especially in endemic regions, is crucial to prevent misdiagnosis and unnecessary surgical or chemotherapeutic interventions. Meticulous analysis of imaging features, such as the nature of ascites, morphology of adnexal lesions, peritoneal thickening patterns, and associated lymphadenopathy, can offer key diagnostic clues. A multidisciplinary diagnostic approach that integrates imaging, ascitic fluid analysis, and targeted biopsies is vital for timely and accurate diagnosis. Tuberculosis should always be considered in the differential, even when imaging findings strongly suggest malignancy.
